# Mortality and transmissibility patterns of the 1957 influenza pandemic in Maricopa County, Arizona

**DOI:** 10.1186/s12879-016-1716-7

**Published:** 2016-08-11

**Authors:** April J. Cobos, Clinton G. Nelson, Megan Jehn, Cécile Viboud, Gerardo Chowell

**Affiliations:** 1 School of Human Evolution and Social Change, Arizona State University, Tempe, AZ USA; 2 School of Life Sciences, Arizona State University, Tempe, AZ USA; 3 Barrett, the Honors College, Arizona State University, Tempe, AZ USA; 4 Division of International Epidemiology and Population Studies, Fogarty International Center, National Institutes of Health, Bethesda, MD USA; 5 School of Public Health, Georgia State University, Atlanta, GA USA

**Keywords:** 1957 influenza, H2N2 virus, Asian influenza, Mathematical epidemiology, Mortality rates, Transmissibility, Reproduction number, Maricopa County, Arizona

## Abstract

**Background:**

While prior studies have quantified the mortality burden of the 1957 H2N2 influenza pandemic at broad geographic regions in the United States, little is known about the pandemic impact at a local level. Here we focus on analyzing the transmissibility and mortality burden of this pandemic in Arizona, a setting where the dry climate was promoted as reducing respiratory illness transmission yet tuberculosis prevalence was high.

**Methods:**

Using archival death certificates from 1954 to 1961, we quantified the age-specific seasonal patterns, excess-mortality rates, and transmissibility patterns of the 1957 H2N2 pandemic in Maricopa County, Arizona. By applying cyclical Serfling linear regression models to weekly mortality rates, the excess-mortality rates due to respiratory and all-causes were estimated for each age group during the pandemic period. The reproduction number was quantified from weekly data using a simple growth rate method and assumed generation intervals of 3 and 4 days. Local newspaper articles published during 1957–1958 were also examined.

**Results:**

Excess-mortality rates varied between waves, age groups, and causes of death, but overall remained low. From October 1959-June 1960, the most severe wave of the pandemic, the absolute excess-mortality rate based on respiratory deaths per 10,000 population was 16.59 in the elderly (≥65 years). All other age groups exhibit very low excess-mortality and the typical U-shaped age-pattern was absent. However, the standardized mortality ratio was greatest (4.06) among children and young adolescents (5–14 years) from October 1957-March 1958, based on mortality rates of respiratory deaths. Transmissibility was greatest during the same 1957–1958 period, when the mean reproduction number was estimated at 1.08–1.11, assuming 3- or 4-day generation intervals with exponential or fixed distributions.

**Conclusions:**

Maricopa County exhibited very low mortality impact associated with the 1957 influenza pandemic. Understanding the relatively low excess-mortality rates and transmissibility in Maricopa County during this historic pandemic may help public health officials prepare for and mitigate future outbreaks of influenza.

## Background

After decades of influenza circulation with relatively low virulence following the 1918 influenza pandemic [1–5], the 1957–58 H2N2 pandemic spread to more than 20 countries in less than 4 months and caused almost 60,000 excess deaths in the United States from September 1957- March 1958 [6–8]. The 1957 influenza pandemic has been associated with an average respiratory excess death rate of 1.9 per 10,000 during 1957–1959 [9]. Moreover, the impact of this pandemic was moderate relative to the 1918 pandemic, but about 10 times greater than the 2009 A/H1N1 influenza pandemic [9].

With the possible exception of persons older than 67 years, individuals had no prior exposure to the A/H2N2 virus, and therefore had no previous immunity to this virus, resulting in about a quarter of the United States population becoming infected [6, 7]. The widespread effects of recent influenza pandemics have emphasized the importance of understanding historical pandemics in order to prepare for and mitigate future outbreaks. A better understanding of the age, seasonal, and transmissibility patterns of previous influenza pandemics may help public health officials prepare for challenges that we may face during future influenza pandemics. Although past studies have quantified the mortality burden of the 1957–58 influenza pandemic in the United States [8, 10–12], little is known about the transmission and mortality characteristics of this pandemic at small spatial scales. Here we aimed to quantify age-specific mortality rates and transmissibility patterns of the 1957–1958 pandemic in Maricopa County using publicly available data comprising a long series of detailed mortality records during 1954–1961.

Originating from the Kweichow province of China in late February 1957, the virus induced fever, sore throat, headache, malaise, and myalgia [13]. The pandemic reached the United States in late May 1957 and the first Asian influenza outbreak in the United States occurred in early June in Newport, Rhode Island [10]. However, the first serologically confirmed case of the H2N2 virus in Arizona was not reported until September 23, 1957 [14].

It has previously been reported that the Mountain region of the United States, which included Arizona, did not experience a second wave of high mortality in early 1958 [10]. However, there is evidence for variation in the excess mortality rates and temporal patterns for different cities and states within the same geographic region [10]. By using publicly available archival death certificates from the Arizona Department of Health Services, we set out to quantify the age-specific seasonal patterns, mortality rates, and transmissibility patterns of the pandemic in Maricopa County and compare our local estimates with those previously derived at the national level.

## Methods

### Geographical setting

Maricopa County, in the south-central region of Arizona, is bordered by mountain ranges on the east, west, and north and includes a portion of the Sonora Desert. While the weather is generally mild during the fall, winter, and spring, temperatures are often above 100 °F (37.8 °C) during the summer. However, the humidity levels generally remain low during the summer months. In mid-summer and early fall, Maricopa County experiences annual monsoons with very heavy rainfall [15]. Although there may be indoor crowding, which may lead to increased disease transmission in the hot summer months and the monsoon season [16], influenza epidemics in Maricopa County are most common during the winter months. As the influenza virus must overcome various environmental factors in order to survive the transport between hosts, climate can be an important factor in the patterns of influenza epidemics [17]. Cases of influenza typically peak in the winter and this may be due to low indoor humidity, cold temperatures, and low solar radiation [16].

Maricopa County consists of 25 cities and towns, the largest of which is Phoenix, the state capital [15]. According to the US Census Bureau, the population of Maricopa County was 331,770 in 1950 and 663,510 in 1960. In 1950, Maricopa County made up 44 % of the total Arizona state population and in 1960 this increased to 51 % [18, 19]. Additionally, the death rate and incidence of tuberculosis in Arizona were notably the highest in the United States in 1957 [20]. Compared to those who lack tuberculosis infection, patients with tuberculosis are more susceptible to influenza infections and more likely to die of influenza, making Maricopa County a particularly interesting location to study influenza because of the relatively high prevalence of tuberculosis in its population during the time period of the influenza pandemic [21, 22].

### Sources of data

Using an online database provided by the Arizona Department of Health Services (http://genealogy.az.gov), a total of 36,585 all-cause archival death records of individual deaths that occurred in Maricopa County between January 1, 1954 and December 8, 1961 were manually retrieved [23]. Death records from 1954–1961 were chosen in order to compare the epidemic period of 1957–1961 with a baseline non-epidemic period during 1954–1956. For each death certificate, the individual’s age at death, gender, exact date of death, and cause(s) of death were recorded from the microfilmed records into a digital spreadsheet. For cause(s) of death, only the presence (1) or absence (0) of influenza, pneumonia, bronco-pneumonia, bronchitis, lung congestion, and tuberculosis were recorded in the spreadsheet. Duplicate death records and addendums to certificates were consolidated into one record. We analyzed three pandemic waves during the 1957–1960 period: October 1, 1957-March 31, 1958; October 1, 1958-June 30, 1959; and October 1, 1959-June 30, 1960.

We grouped the individuals into 6 age categories (<5, 5–14, 15–24, 25–44, 45–64, ≥65 years) and assembled weekly and monthly time series for deaths from respiratory illnesses as well as all-causes. We chose to use narrow age categories to enable greater precision when comparing different age groups as well as more resolution on the specific age groups most affected by the pandemic. Influenza, pneumonia, bronco-pneumonia, bronchitis, and lung congestion were categorized as respiratory causes of death. It has previously been suggested that the severity of influenza epidemics cannot be fully measured by influenza and pneumonia deaths [8, 10, 24]. The magnitude of influenza epidemics is better represented by measuring the total excess mortality from all causes or the excess mortality due to all respiratory causes [8, 24]. As influenza infections are known to occur concurrently with other respiratory illnesses and may not be diagnosed as influenza, pneumonia, bronco-pneumonia, bronchitis, and lung congestion cases were also abstracted in addition to influenza [25, 26]. Due to the high prevalence of tuberculosis in Arizona, tuberculosis deaths were excluded from the excess mortality, standardized mortality ratio, and reproduction number calculations based on respiratory causes. Information about age, gender, exact date of death, and/or cause of death were not available for <1 % of all records.

### Qualitative data

To provide anecdotal references about the course of the pandemic in Maricopa County during 1957–1958, we examined the most popular daily newspaper of the area, *The Arizona Republic*, which was published in Phoenix, Arizona. From June 1957-March 1958, there were 65 articles referencing influenza, 12 of which included news of Maricopa County. These archival newspaper articles were manually retrieved from the Arizona State University microform library in Tempe, Arizona. This data was used to create a timeline of relevant events and non-pharmacological mitigation strategies employed in Arizona during the pandemic period.

### Statistical methods

#### Population estimates

Although the US Census Bureau provided detailed population data every decade, sufficient Maricopa County population data were not available for 1954–1959 and 1961. As the Maricopa County population almost doubled between 1950 and 1960, we estimated the population values for the intercensal years of 1954–1959 and 1961. To estimate the total Maricopa County population for each week from January 3, 1954 to December 3, 1961, a polynomial model was applied to county population data provided by the 1950 and 1960 United States Censuses as well as Valley National Bank annual January intercensal county estimates spanning from 1951 through 1959 and 1961 [18, 19, 27].

Using the total and age-specific county population data from the 1950 and 1960 United States Censuses, we calculated the proportion of each age-specific population to the total county population for 1950 and 1960 and used polynomial models to estimate the age-specific to total county population proportions for each week from January 3, 1954 to December 3, 1961. These estimated age-specific to total county population proportions for each week were then multiplied by the previously estimated total Maricopa County population for the same week to estimate the weekly age-specific population values from January 3, 1954 to December 3, 1961. The proportion and final weekly age-specific population estimation steps were repeated for each age category. These age-specific Maricopa County population estimates were used to calculate age-specific mortality values. To represent the estimated total county population for each week, all the final age-specific estimations were summed for that week and these sums were used to calculate mortality values for all-ages.

Instead of directly estimating the weekly age-specific population values from the two data points of the 1950 and 1960 U.S. censuses, this procedure was chosen in order to account for the gradual change in age-specific proportions while including the additional data from the Valley National Bank intercensal county estimates to create a more representative model. Additionally, this method accounts for specific deviations between age groups as well as for changes in the age structure of the population by including the age-specific populations of 1950 and 1960 in the models.

#### Mortality data

To represent the mortality rate linked to the 1957–1958 influenza pandemic in Maricopa County, we quantified the excess mortality per 10,000 people for each age category and each expected pandemic wave. For each expected wave and age group, excess mortality was defined as the number of deaths during the pandemic period greater than the baseline mortality from a comparable time period without epidemic influenza (Excess Mortality = mortality during epidemic period- baseline mortality) [10]. Weekly mortality data for the pre-pandemic period of January 3, 1954-June 30, 1957 and a cyclical Serfling linear regression model, including temporal trends and harmonic terms for seasonality, were utilized to estimate the baseline mortality [28, 29]. Based on a time series of monthly respiratory mortality rates of all-ages, three expected pandemic waves were chosen for excess mortality analysis: October 1, 1957-March 31, 1958, October 1, 1958-June 30, 1959, and October 1, 1959-June 30, 1960. The model used to estimate baseline-mortality was expressed as: weekly death rates(t) = intercept + α_1_* t + α_2_(t/100)^2^ + α_3_(t/100)^3^ + α_4_(t/100)^4^ + β_1_sin(2*π/52.17*t) + γ_1_cos(2*π/52.17*t) + β_2_sin(4*π/52.17*t) + γ_2_cos(4*π/52.17*t) + β_3_sin(8*π/52.17*t) + γ_3_cos(8*π/52.17* t), where t represented the week number and α, β, and γ were coefficients to be estimated from the data. In the above model, α represented the time trend and β and γ represented seasonal changes. The baseline-mortality model was adapted from Chowell et al. [28].

During each expected wave, pandemic periods were classified as weeks with respiratory or all-cause mortality above the 95 % upper confidence limit (UCL) of the baseline mortality. Weekly excess mortality was equal to the number of deaths greater than the baseline model during these pandemic periods. To find the absolute mortality burden of each expected pandemic wave in 1957–1960, the excess deaths greater than the baseline mortality were summed during each pandemic period [2, 29]. Separate models with age-specific population values and age-specific weekly deaths were fit to each age category for both respiratory deaths and all-causes.

Additionally, we calculated the ratio of observed mortality during the pandemic periods to the expected baseline mortality of a period lacking pandemic influenza, or the standardized mortality ratio (SMR) for pandemic-related death. To estimate the mortality attributable to the influenza pandemic, we calculated mortality rate in excess of a seasonal model baseline. The expected baseline mortality for a period lacking pandemic influenza was estimated using the cyclical Serfling linear regression model previously described as well as mortality data from the pre-pandemic period from January 3, 1954 to June 30, 1957. These mortality ratios have been standardized by the same age categories used for excess mortality. Standardized mortality ratio is interpreted relative to 1, with values greater than 1 representing an increased risk of death in individuals exposed to the H2N2 virus compared to individuals who were unexposed.

#### Reproduction numbers (transmission characteristics)

For each expected pandemic wave, we estimated the intrinsic transmission factor. At the beginning of an epidemic, the transmission factor of a pathogen is measured by the basic reproduction number (R_0_), the average number of secondary cases generated by a primary case in a completely naive population [2, 30, 31, 34]. However, as the outbreak continues, the population is no longer completely susceptible due to adaptive immunity [32]. In a partially immune population, the transmission potential during the initial epidemic period is defined as the reproduction number (R). As there is slight or no background population immunity during the initial wave of a pandemic, R is expected to approximately equal R_0_ during the beginning of the first wave. However, based on the season that the new virus was introduced to local populations, the reproduction number may vary geographically and temporally [2].

A growth rate method was used to estimate the reproduction number. The growth rate (*r*) measures how quickly the number of cases increases through time and is estimated by assuming an exponential function to the initial increase in the weekly respiratory deaths. A straight line can be modeled to the data through taking the log of weekly deaths during the ascending phase, using the following regression: log(*weekly cases*(*t*)) = *intercept* + *r* ∗ *t*, where t = a daily index and r = an estimated regression coefficient that represents the exponential growth rate.

The ascending phase was defined as the time between the week the pandemic began, the first week of the period with continuously increasing deaths, and the week the wave peaked. Based on the Susceptible-Exposed-Infectious-Recovered transmission model, the reproduction number was calculated by substituting *r* into the following equation: $R=\left(1+\frac{r}{b_1}\right)\left(1+\frac{r}{b_2}\right)$ where $\frac{1}{b_1}$ =mean latent period and $\frac{1}{b_1}$ = mean infectious period. In the previous equation for R, the latent and infectious periods are assumed to be exponentially distributed and the mean generation interval is found by ${T}_C=\frac{1}{b_1}+\frac{1}{b_2}$.

Additionally, an upper bound estimate in the case of a fixed generation interval (delta distribution) was obtained with the following equation: $R={e}^{r{T}_C}$.

As the generation interval for influenza is uncertain, two generation intervals were used. A short generation interval of 3 days, with a latency period of 1.5 days and an infectious period of 1.5 days, was used. Additionally, a longer generation interval of 4 days, with a latency period of 2 days and an infectious period of 2 days, was used. The generation interval measures the length of time between when symptoms develop in 2 consecutive cases and is made up by the infectious period of the initial case and the latency period of the second case [2, 28, 33].

## Results

### Local newspapers, 1957–1958

**Fig. 1:**
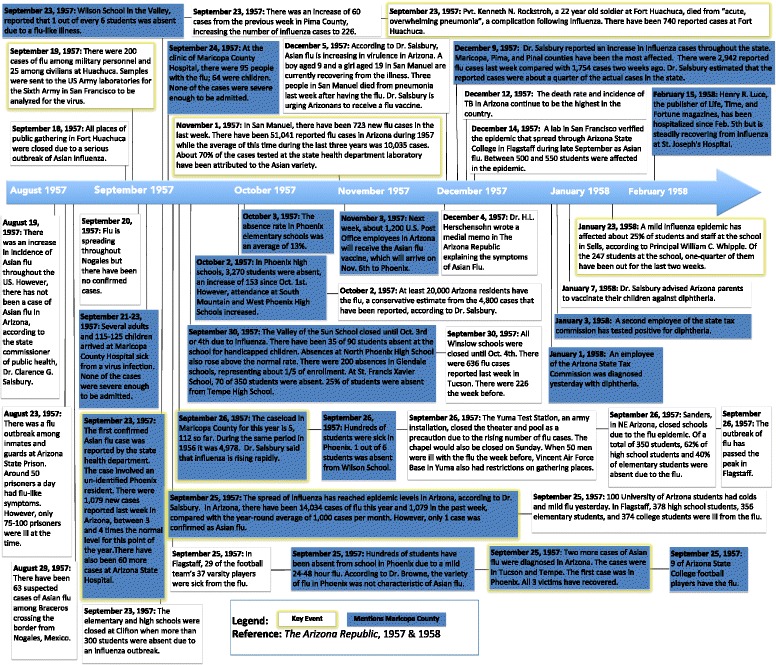
Timeline of Events in Arizona. A summary of the major events documenting the severity and spread of influenza and other respiratory illnesses in Arizona during the introduction of the H2N2 virus, based on articles in *The Arizona Republic* from June 1957-March 1958. For a more complete set of articles see https://www.dropbox.com/sh/irz1zzf8z613p8j/AACLkXzyXikWqskIssRv7aBha?dl=0

The first article in The Arizona Republic of an influenza outbreak in Arizona appeared on August 24, 1957 when about 75–100 prisoners at Arizona State Prison in Pinal County had flu-like symptoms (see Fig. 1) [35]. On September 19, 1957 another outbreak was reported from Fort Huachuca in Cochise County and all places of public gathering were closed due to 225 cases of influenza, unconfirmed as the H2N2 strain [36, 37]. After its introduction into Arizona, the influenza virus spread rapidly throughout the state. The first confirmed case of “Asian” influenza in Arizona, which involved a Phoenix resident, was reported on September 23, 1957 [14]. There had been 14,034 cases of influenza reported in Arizona from the beginning of the year to September 25, 1957, when the transmission of the virus reached epidemic levels, according to the state health commissioner [38]. In Maricopa County specifically, there had been 5112 cases of influenza during the year by September 26, 1957 and influenza cases continued to rise rapidly [39].

On December 8, 1957, the state health commissioner reported an increase in influenza cases throughout the state, with Maricopa County being one of the most affected regions. In the week prior to his announcement, there had been 2942 cases of influenza, representing about a quarter of the actual cases in the state [40]. As time progressed into early 1958, there were fewer reports on influenza in Maricopa County and Arizona.

### Excess mortality and standardized mortality ratio attributable to influenza

**Fig. 2:**
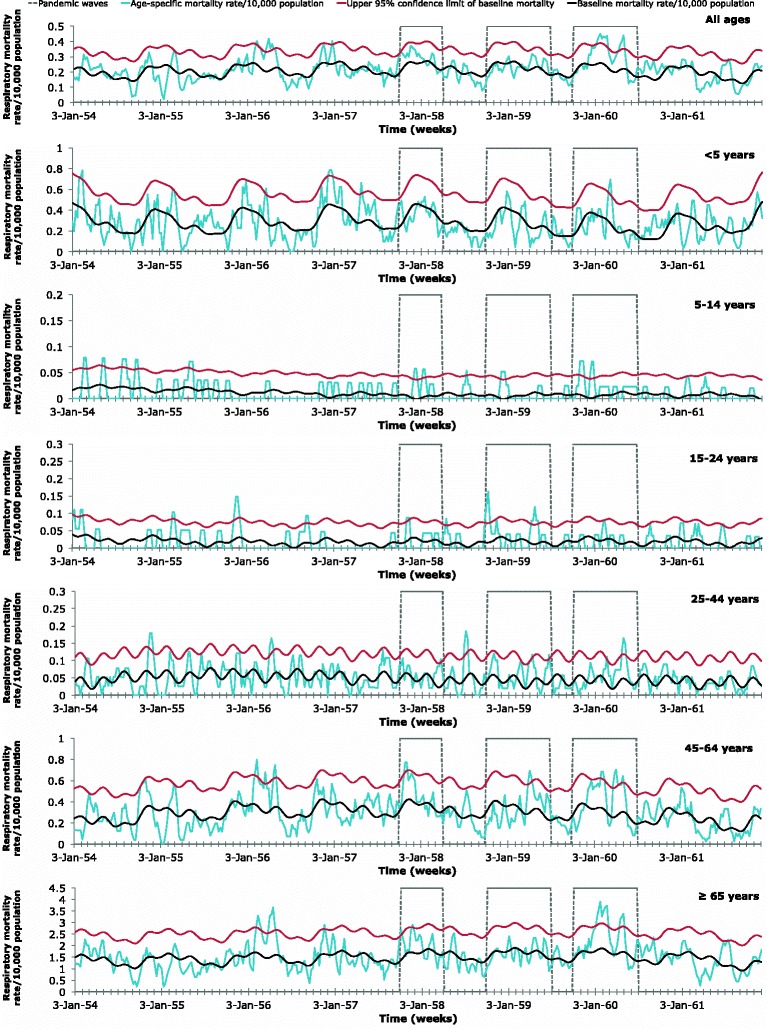
Age-specific respiratory mortality weekly time series. 2 Age-specific weekly time series of respiratory mortality per 10,000 population in Maricopa County, Arizona, 1954–1961. Areas outlined in gray represent the three expected pandemic waves: October 1, 1957-March 31, 1958; October 1, 1958-June 30, 1959; and October 1, 1959-June 30, 1960. The baseline mortality (*black*) was estimated using a cyclical Serfling linear regression model. The baseline mortality’s 95 % upper confidence limit (UCL) is also shown (*red*). Mortality attributable to the 1957 influenza pandemic was defined as the mortality rates (*blue*) in excess of the baseline mortality, when the mortality rates exceeded the 95 % UCL of the baseline mortality during the expected pandemic waves

**Fig. 3:**
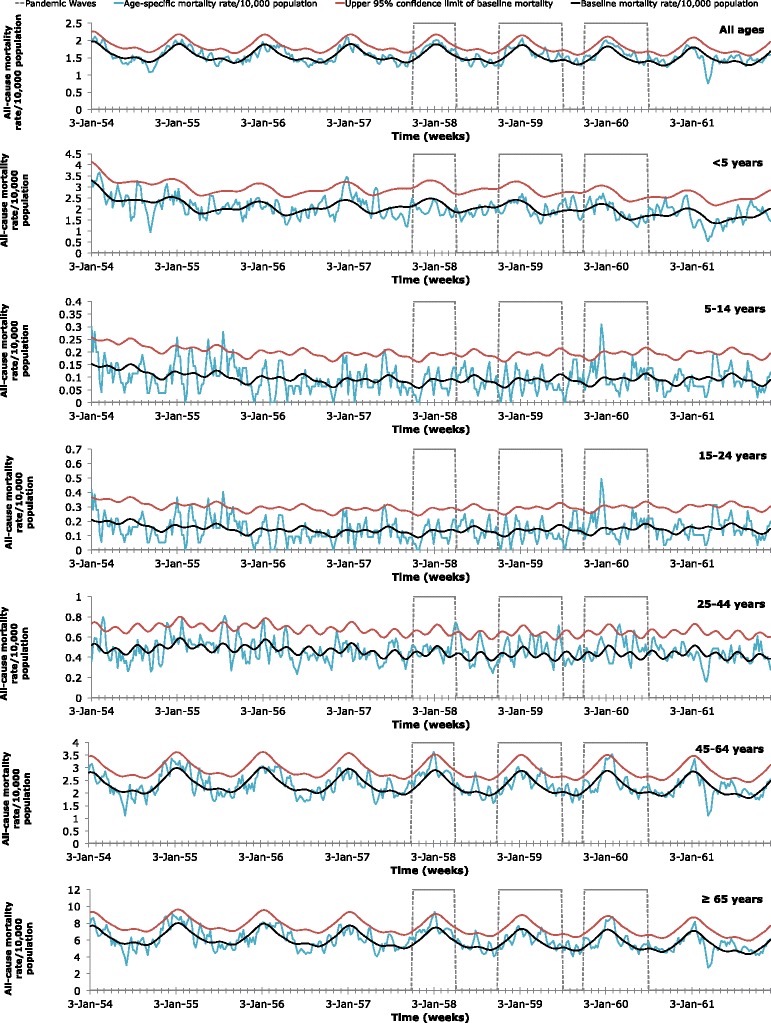
Age-specific all-cause mortality weekly time series. Age-specific weekly time series of all-cause mortality per 10,000 population in Maricopa County, Arizona, 1954–1961. Areas outlined in gray represent the three expected pandemic waves: October 1, 1957-March 31, 1958; October 1, 1958-June 30, 1959; and October 1, 1959-June 30, 1960. The baseline mortality (*black*) was estimated using a cyclical Serfling linear regression model. The baseline mortality’s 95 % upper confidence limit (UCL) is also shown (*red*). Mortality attributable to the 1957 influenza pandemic was defined as the mortality rates (*blue*) in excess of the baseline mortality, when the mortality rates exceeded the 95 % UCL of the baseline mortality during the expected pandemic waves

Mortality from 1957–1961 was extremely mild in Maricopa County, compared to previous influenza pandemics. The respiratory mortality weekly time series for all-ages in Maricopa County demonstrated evidence only of two mild pandemic waves during the 1957–1960 pandemic time period: a 7 week period from January 10, 1960 until February 28, 1960 and a 3 week period from April 17, 1960 until May 8, 1960 (Fig. 2). Based on all-causes, there were three short periods of excess mortality for all-ages: a 1-week period from November 9, 1958 until November 16, 1958, a 1-week period from April 19, 1959 until April 26, 1959, and 1-week period from May 1, 1960 until May 8, 1960 (Fig. 3).

The absolute excess-mortality rates per 10,000 population for each predicted wave were summed for respiratory illnesses (Table 1) and all-causes (Table 2). In general, absolute excess mortality remained very low in those younger than 24 and increased with age in deaths due to all-causes, with some variation between waves. In absolute excess mortality due to respiratory causes, there was more variation with age. With the exception of respiratory causes during the 1958 wave, absolute excess-mortality for respiratory or all-causes peaked among the elderly (≥65 years).

**Table 1:** Estimated age-specific absolute excess mortality rates and standardized mortality ratios for respiratory causes of death, Maricopa County^a^

	1957		1958		1959	
Age Group (yrs)	Excess mortality rate/10,000 population	SMR	Excess mortality rate/10,000 population	SMR	Excess mortality rate/10,000 population	SMR
All ages	0.00	1.17	0.00	1.01	1.80	1.31
<5	0.00	0.95	0.00	0.85	0.75	1.04
5–14	0.22	4.06	0.16	1.37	0.38	3.28
15–24	0.19	1.16	0.70	1.88	0.00	0.81
25–44	0.08	1.24	0.23	1.40	0.38	1.40
45–64	0.72	1.23	1.01	1.02	2.48	1.32
≥65	2.52	1.18	0.00	0.99	16.59	1.36

^a^Absolute excess mortality rates/10,000 population based a cyclical Serfling linear regression model and weekly respiratory mortality rates during the three expected pandemic waves: October 1, 1957-March 31, 1958 (1957), October 1, 1958-June 30, 1959 (1958), and October 1, 1959-June 30, 1960 (1959)

**Table 2:** Estimated age-specific absolute excess mortality rates and standardized mortality ratios for all-causes of death, Maricopa County^a^

	1957		1958		1959	
Age Group (yrs)	Excess mortality rate/10,000 population	SMR	Excess mortality rate/10,000 population	SMR	Excess mortality rate/10,000 population	SMR
All ages	0.00	1.05	0.64	1.03	0.31	1.06
<5	0.00	0.87	0.00	0.98	0.00	1.01
5–14	0.00	0.90	0.00	0.85	0.48	1.25
15–24	0.00	0.91	0.00	0.85	1.08	1.24
25–44	0.53	1.08	0.77	1.15	1.06	1.11
45–64	0.74	1.10	1.41	1.02	0.73	1.03
≥65	1.85	1.09	3.50	1.02	1.73	1.02

^a^Absolute excess mortality rates/10,000 population based a cyclical Serfling linear regression model and weekly all-cause mortality rates during the three expected pandemic waves: October 1, 1957-March 31, 1958 (1957), October 1, 1958-June 30, 1959 (1958), and October 1, 1959-June 30, 1960 (1959)

**Fig. 4:**
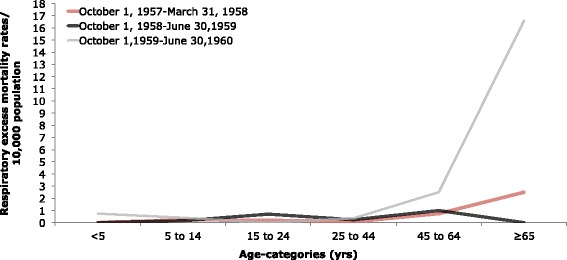
Age-specific absolute respiratory excess mortality rates/10,000 population. Age-specific absolute excess-mortality rates per 10,000 population during the three expected pandemic waves (October 1, 1957-March 31, 1958; October 1, 1958-June 30, 1959; and October 1, 1959-June 30, 1960) of the 1957 pandemic in Maricopa County, Arizona based on deaths attributed to respiratory illnesses. Estimates were in excess of baseline mortality rates for a period with non-epidemic influenza based on a cyclical Serfling linear regression model and weekly respiratory mortality rates

**Fig. 5:**
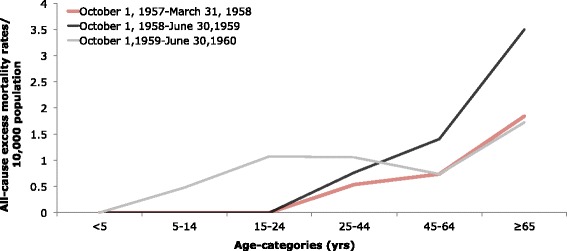
Age-specific absolute all-cause excess mortality rates/10,000 population. Age-specific absolute excess-mortality rates per 10,000 population during three expected pandemic waves (October 1, 1957-March 31, 1958; October 1, 1958-June 30, 1959; and October 1, 1959-June 30, 1960) of the 1957 pandemic in Maricopa County, Arizona based on deaths attributed to all-causes. Estimates were in excess of baseline mortality rates for a period with non-epidemic influenza based on a cyclical Serfling linear regression model and weekly all-cause mortality rates

The absolute excess-mortality rates per 10,000 population for each predicted wave were compared for respiratory illness (Fig. 4) and for all-causes (Fig. 5).In a comparison of the three predicted waves, the greatest absolute excess mortality rate based on respiratory illnesses was observed in those over 65 years of age during the 1959 predicted wave. The 1959 predicted wave demonstrated relatively high absolute excess morality due to respiratory causes in older populations but low values in those younger than 44, with a slight elevation at those younger than 5 years.

For absolute excess mortality based on all-causes, the highest value in a comparison of the three predicted waves was observed during the 1958 wave in those ≥65 years. In all predicted waves, the ≥65 age group experienced the greatest absolute excess-mortality due to all-causes.

To better compare different age groups, which have different background risks of death, the risk for mortality rates relative to baseline-mortality rates were calculated for respiratory causes (Table 1) and all-causes (Table 2). While absolute excess-mortality was generally greatest among the elderly, the standardized mortality ratio was greatest in children (5–14 years) for respiratory causes. For children and young adolescents (5–14), mortality rates increased 4.06-fold above baseline-mortality rates for respiratory causes during the 1957 wave. For all-causes, the standardized mortality ratio was greatest in children (5–14) in the 1959 expected wave, when mortality rates increased 1.25-fold above baseline-mortality rates. For all-ages, there were 1.80 excess respiratory deaths during all three predicted waves.

### Reproduction numbers (transmission characteristics)

Estimates for the reproduction number, based on growth in weekly respiratory death rates, for each predicted wave of the 1957 pandemic in Maricopa County are listed in Table 3. The 1957 wave had the greatest value for R with 1.08, using a short generation interval of 3 days, and 1.10–1.11, using a longer generation interval of 4 days.

**Table 3:** Mean estimates of the reproduction number (R) and 95 % confidence levels due to respiratory causes^a^

	3-day generation interval		4-day generation interval	
Wave	Exp. distribution	Delta distribution	Exp. distribution	Delta distribution
1957	1.08 (0.99, 1.17)	1.08 (0.99, 1.18)	1.10 (0.99, 1.23)	1.11 (0.99, 1.24)
1958	1.05 (1.00, 1.11)	1.05 (1.00, 1.11)	1.07 (1.00, 1.14)	1.07 (1.00, 1.15)
1959	1.05 (1.01, 1.08)	1.05 (1.01, 1.08)	1.07 (1.02, 1.11)	1.07 (1.02, 1.12)

^a^Values were estimated from weekly data based on three expected pandemic waves: October 1, 1957-March 31, 1958 (1957), October 1, 1958-June 30, 1959 (1958), and October 1, 1959-June 30, 1960 (1959). A generation interval of 3 or 4 days was assumed, with an exponential (exp.) or fixed (delta) distribution

## Discussion

By analyzing primary data from archival death certificates from 1954 to 1961 and archival newspaper articles, we found that Maricopa County exhibited low mortality impact associated with the 1957 influenza pandemic, compared with other regions of the United States. In the United States, excess mortality values for all-ages from pneumonia and influenza deaths as well as from all-cause deaths were greatest from October-December 1957, compared to January-March 1958 and January-March 1960 [11]. From September 1957 to March 1958, the US had a 4.5 (per 10,000) absolute all-cause excess mortality value for all ages and a 1.17 (per 10,000) absolute influenza-pneumonia excess mortality value for all ages [10]. However, in Maricopa County, the absolute respiratory excess mortality for all-ages was greatest (1.8 per 10,000) during the 1959–1960 wave and excess mortality peaked in the first week of May 1960. While some age groups did have extremely mild excess-mortality during October 1, 1957-March 31, 1958 or during October 1, 1958-June 30, 1959, there was little overall evidence for herald waves in 1957 or 1958, based on respiratory deaths. This is consistent with the less pronounced mortality from January-March 1958 and the higher excess mortality from 1959 to 1960 in the Mountain region compared to other regions of the U.S. [11]. Absolute all-cause excess mortality (per 10,000) in Maricopa County was greatest from October 1, 1958-June 30, 1959, for all-ages (0.64) and for the elderly (≥65) (3.50). However, absolute excess-mortality from all-causes was minimal throughout all predicted waves. Interestingly, there was also some excess-mortality from respiratory or all-causes during the week of June 2, 1957 or during June 29, 1958-July 20, 1958 for some age groups, but not overall. We cannot rule out the potential contribution of high temperature in the region to excess mortality during these summer months. It has also been suggested that influenza epidemics may coincide with rainy reasons in the tropics due to indoor crowding [16]. However, influenza transmission in Maricopa County seems to be more efficient in cold, dry conditions with low air pressure. Soebiyanto et al. also demonstrated that influenza cases in Maricopa County do not seem to be associated with rainfall [41].

The virus seemed to have been introduced in Maricopa County relatively late, between August 23, 1957 and September 23rd, 1957 [14, 35]. The U.S. had seen its first case by June and the first epidemic in the U.S. occurred in early August in the southeast of the U.S. [6, 13]. However, the first case of the H2N2 virus in Arizona was not confirmed until September 23, 1957 and transmission reached epidemic levels in Arizona on September 25, 1957 [14, 38]. One of the major factors for the emerging community epidemics in the fall of 1957 was the opening of schools around September [6]. Mortality began to rise during the fourth week of October 1957, about 3 weeks after the rest of the U.S. [11]. Mortality in Maricopa County peaked in the third week of November 1957, about 2 weeks after other regions of the U.S. [11]. According to newspaper reports, influenza incidence in Maricopa County was still rising rapidly on September 26, 1957 and seemed to continue to rise at least through November 1, 1957 [39, 42]. A rise in mortality can follow a rise in acute respiratory illness incidence by as much as 3–4 weeks [11]. This lag between morbidity and mortality may be because the 1957 influenza virus generally affected high school age adolescents first, followed by elementary school students, and the adult population last. [13]. Incidence in the United States was especially high in those between 5 and 19 and lowest in those 65 and older. However, mortality was highest in those 65 and older [13]. Our results confirm that age-patterns in Maricopa County were similar to those from the rest of the United States, with the excess-mortality concentrated in the elderly (≥65). While incidence may have been high in children and adolescents, this age group experienced minimal excess-mortality, avoiding the effects of an over-reactive immune system and the over-production of cytokines theorized for the high mortality rates of young adults reported during the 1918 pandemic [43]. Instead, younger individuals may have transmitted the virus to the elderly after a couple of weeks of high incidence in school-aged populations. While individuals >67 years of age may have had antibodies for the 1957 H2N2 virus due to a possibly related pandemic in 1889–90, individuals ≥65 years of age also had a high-risk of death from influenza in 1957 due to cardiovascular disease and bronco-pulmonary co-morbidities [8, 44].

From October 1957-March 1958, excess deaths from all-causes in the United States were greatest in the elderly (≥65) and demonstrated a U-shaped age-pattern (high mortality in infants and elderly with low mortality in young adults). While all-cause excess deaths were concentrated in those 65 and older, there were no all-cause excess deaths in infants (≤1 year) and low all-cause excess deaths in children (1–14 years), avoiding the U-shaped age pattern in the United States from January-March 1960 [11]. With the exception of excess-mortality rates from respiratory causes during October 1, 1958-June 30, 1959, excess-death rates in Maricopa County were greatest in the elderly and had minimal values in younger age groups. While other periods demonstrated no excess-mortality in young children (≤5), the 1959–1960 wave had a very slight elevation in excess-mortality for the age group. However, the excess-mortality in those less than 5 years did not approach that of the elderly, as is common in a traditional U-shaped age-pattern. Therefore, the age-pattern did not truly resemble a U-shaped curve.

For both all-causes and respiratory illnesses, the standardized mortality ratios were minimal for most age groups throughout all three waves. Most likely due to crowding in schools, the standardized mortality ratio peaked (4.06) in young children and adolescents (5–14 years) from October 1, 1957-March 31, 1958, based on mortality rates of respiratory deaths. This is consistent with what was reported by Dauer: the epidemic in September 1957 began in high schools and colleges and moved into elementary schools and pre-school children. However, in the United States from October-December 1957, the standardized mortality ratio was highest (~2.25) in those 30–39 years old, perhaps due to proximity in the workplace [10]. Additionally, it is important to note that although the standardized mortality ratio was elevated for children and adolescents during the 1957 wave, the excess-mortality rate was minimal for the same age group and time period. However, these results are not contradictory. While the baseline mortality was a fraction of the observed mortality during the 1957 wave, the difference between the values was negligible for young children and adolescents. This may be due to a relatively low baseline mortality in those aged 5–14, compared to other age groups. In contrast, the higher excess mortality rate and the low SMR seen in those ≥65 during the 1959 wave may be due to a higher baseline mortality for the elderly, when compared to other age-categories.

Although the respiratory excess-mortality rates during the 1957 and 1959 waves both disproportionally affected the elderly, there was a shift to greater excess mortality in the latter wave. A previous study showed that excess-mortality during the 1959 wave was concentrated in the elderly and approached the excess-mortality rate of the 1957 wave [11]. While excess-mortality may have increased between waves in Maricopa County, the possibility that the 1959 wave could have been due to a different influenza strain cannot be ruled out. This study however did not address evidence demonstrating that the 1959–1960 dominant strain in Maricopa County was the 1957 pandemic strain.

To estimate the baseline mortality from a non-epidemic period, this study utilized mortality data from January 3, 1954 to June 30, 1957. Baseline periods vary in length between studies and longer periods may be used for country-wide analyses. Using a longer period to estimate baseline mortality may have increased the accuracy of the Serfling model. However, a three-year period has been previously used to estimate the epidemic threshold of smaller populations, such as cities or counties, in which there is reduced variation [2].

Our calculation of excess mortality is not exempted of limitations. In particular, due to lack of laboratory confirmation, our excess mortality approach would not have been unable to distinguish elevation in mortality rates associated with other causes and coinciding with the pandemic period. Our approach for calculating excess mortality was relatively simple, due to lack of contemporaneous virological surveillance. Moreover, by grouping deaths into all-cause mortality and respiratory mortality categories, the study prioritized sensitivity over specificity. Because influenza deaths are often attributed incorrectly, we believe that categorizing deaths into all-causes and respiratory causes provides conservative estimates of excess mortality.

Reproduction numbers were relatively similar between waves, assuming mean generation intervals of 3 or 4 days that follow exponential or fixed distributions. In the United Kingdom, R_0_ was estimated to be 1.7–1.8 for an infectious period of 2 days and 1.5–1.6 if the infectious period was 1.5 days [45]. Thus, the Maricopa County mean reproduction number of 1.08–1.11, using 3 or 4 day generation intervals and exponential or fixed distributions, was substantially lower than that observed in the UK. However, the reproduction number is preferably calculated from case incidence curves rather than time series of deaths. Consequently, it is likely that our R_0_ estimates could be slightly underestimated. Nevertheless, the lower reproduction number observed in Maricopa County in the 1957–1958 pandemic wave was most likely not due to the public health interventions put in place in the fall of 1957. While closing schools can reduce the effects of an epidemic by 22 %, when the R_0_ is low (≤1.8) [45], the state health department seemed to implement few non-pharmacological mitigation strategies, and instead urged residents to receive an influenza vaccine and communicated the symptoms of Asian flu [46, 47]. While Valley of the Sun School closed for about 5 days on September 30th, when absences reached 39 % of enrollment, most schools remained opened [48]. Absences reached 25 % of enrollment in Glendale schools, 20 % at St. Francis Xavier School, and 25 % at Tempe High School [48]. However, absences in Phoenix elementary schools in the first week of October were only 13 % of enrollment [49].

While some have theorized that the 1918 and 1957 differed in virulence, the differences in the rates of severe disease are likely due to medical and public health advances. In 1918–19, almost all of the well-observed influenza deaths were due to bacterial infections in the lungs [50]. Similarly, in 1957, deaths were often associated with bacterial pneumonia and staphylococcal infections [51]. However, unlike the 1918 pandemic, secondary bacterial infections were partially controlled through antimicrobials in 1957 [7]. The bacterial infections that did result in death were generally multidrug resistant [52]. 1957 was the first time a pandemic virus was available for laboratory analysis and the first time that an influenza vaccine became available [7, 50, 52]. Although 22,017 vaccine doses were reportedly allocated to Arizona, newspaper reports suggest that many of these doses had not been received by November 1957 [38, 53] casting doubt on the explanation that low mortality rates in Maricopa County might be explained by vaccine administration.

Climate and its effects on virus survival and transmission may be a possible explanation for the lower mortality rates and reproduction numbers observed in Maricopa County during the expected waves. Influenza virus survival is optimal at low temperatures, low sunlight, and low absolute humidity [16]. However, while humidity was generally low in Maricopa County, temperature and sunlight during the winter were not low, compared with other regions of the U.S. These environmental conditions may have contributed to the decreased reproduction number and mortality rates, as it would have been more difficult for the virus to survive transmission between hosts.

## Conclusions

By using primary data from archival death certificates from 1954 to 1961 to quantify the age, seasonal, and transmissibility patterns of the second influenza pandemic of the 20th century, this study confirmed that Maricopa County largely avoided the effects of the 1957 pandemic. Compared to other regions of the United States, Maricopa County had few excess deaths due to the 1957 influenza pandemic. Although results varied between age groups, the 1957 pandemic in Maricopa County was characterized by a mild wave from October 1, 1959 to June 30, 1960, when there were 16.59 absolute excess-deaths due to respiratory causes per 10,000 population in the elderly (≥65 years), the age group most affected. However, the standardized mortality ratio peaked (4.06) in children and young adolescents (5–14 years) from October 1, 1957-March 31, 1958, based on mortality rates of respiratory deaths. Transmissibility was greatest during the same 1957–1958 period, when the mean reproduction number was a low 1.08–1.11, using 3 or 4-day generation intervals and exponential or fixed distributions.

Through analyzing archived newspaper articles, the low mortality and transmissibility rates recorded in Maricopa County were most likely not due to public health interventions or vaccine distribution. While there is much unknown about climate and virus survival, the environmental conditions in Maricopa County may have prevented high transmission and excess-mortality rates. By analyzing historical data of different regions, researchers can better understand how mortality and transmission rates are related to different environmental conditions and public health interventions, providing important lessons to optimize current country-level preparedness and control plans.

## Abbreviations

R, Reproduction number; R_0,_ Basic reproduction number; SMR, Standardized mortality ratio; UCL, Upper confidence limit; *r*, Growth rate
